# Deficiency of Human Adenosine Deaminase Type 2 – A Diagnostic Conundrum for the Hematologist

**DOI:** 10.3389/fimmu.2022.869570

**Published:** 2022-05-03

**Authors:** Rakesh Kumar Pilania, Aaqib Zaffar Banday, Saniya Sharma, Rajni Kumrah, Vibhu Joshi, Sathish Loganathan, Manpreet Dhaliwal, Ankur Kumar Jindal, Pandiarajan Vignesh, Deepti Suri, Amit Rawat, Surjit Singh

**Affiliations:** Pediatric Allergy Immunology Unit, Department of Pediatrics, Advanced Pediatrics Centre, Post Graduate Institute of Medical Education and Research (PGIMER), Chandigarh, India

**Keywords:** deficiency of human adenosine deaminase type 2, haematological abnormalities, inborn errors of immunity (IEIs), lymphoproliferation, bone marrow failure syndromes, cytopenia

## Abstract

Deficiency of adenosine deaminase type 2 (DADA2) was first described in 2014 as a monogenic cause of polyartertitis nodosa (PAN), early onset lacunar stroke and livedo reticularis. The clinical phenotype of DADA2 is, however, very broad and may involve several organ systems. Apart from vasculitis, children may present with i) Hematological manifestations (ii) Lymphoproliferation and iii) Immunodeficiencies. Patients with DADA2 can have variable patterns of cytopenias and bone marrow failure syndromes. Patients with DADA2 who have predominant haematological manifestations are associated with ADA2 gene variants that result in minimal or no residual ADA2 activity. Lymphoproliferation in patients with DADA2 may range from benign lymphoid hyperplasia to lymphoreticular malignancies. Patients may present with generalized lymphadenopathy, splenomegaly, autoimmune lymphoproliferative syndrome (ALPS) like phenotype, Hodgkin lymphoma, T-cell large granular lymphocytic infiltration of bone marrow and multicentric Castleman disease. Immunodeficiencies associated with DADA are usually mild. Affected patients have variable hypogammaglobulinemia, decrease in B cells, low natural killer cells, common variable immunodeficiency and rarely T cell immunodeficiency. To conclude, DADA2 has an extremely variable phenotype and needs to be considered as a differential diagnosis in diverse clinical conditions. In this review, we describe the evolving clinical phenotypes of DADA2 with a special focus on haematological and immunological manifestations.

## 1 Introduction

Deficiency of adenosine deaminase 2 (DADA2) is a multifaceted autosomal recessive autoinflammatory syndrome. It is caused by loss-of-function homozygous or compound heterozygous variants in *ADA2* (*adenosine deaminase 2*) gene, formerly named as *CECR1* (*cat eye syndrome chromosome region, candidate gene 1*) (1, 2). Initial descriptions of this disorder were published in 2014 with early-onset stroke, recurrent bouts of inflammation, and familial vasculopathy resembling polyarteritis nodosa (1, 2). Phenotypic descriptions of DADA2 have been expanded considerably and now include vasculopathy, lymphoproliferation, immunodeficiency and bone marrow dysfunction. The large phenotypic variability makes DADA2 a true multisystemic and multifaceted disorder. It is possible that several other phenotypic presentations of DADA2 is due for recognition in coming future.

Hematological presentations of DADA2 including immune cytopenias and lymphoproliferation (both benign as well as lymphohematopoietic neoplasms) are increasingly being recognized (3). Establishing a diagnosis of DADA2 in patients with hematological disorders is imperative due to immense therapeutic and prognostic implications. We herein review the diverse clinical spectrum of DADA2 with special focus on hematological manifestations.

## 2 Pathophysiology of ADA Deficiency

In humans, two types of partially homologous adenosine deaminase (ADA) enzymes (ADA1 and ADA2) regulate purine metabolism, converting adenosine/2’-deoxyadeosine to inosine/2’-deoxyinosine. While ADA1 is monomeric and predominantly intracellular, ADA2 is the secreted isoform which also exists as dimers.

### 2.1 ADA1 Deficiency

Though ADA1 is expressed in all human tissues, maximal expression is noted in lymphocytes and is critically important for development of adaptive immune system (4–6). Deficiency of ADA1 results in severe combined immunodeficiency with profound depletion of T, B, and NK cells due to accumulation of toxic deoxyadenosine nucleotides.

### 2.2 ADA2 Deficiency

ADA2 is highly expressed in myeloid cells and is secreted by activated macrophages, monocytes and dendritic cells (7–9). ADA2 also interacts with lymphocytes and other leukocytes through adenosine receptors (10). ADA2 plays a significant role in development of hematopoietic and endothelial cells, maintaining balance between M1 and M2 macrophages (7, 9, 11). Pathophysiology of DADA2 is still evolving. In DADA2, monocyte differentiation is skewed towards pro-inflammatory M1 macrophages and results in generation of inflammatory cytokines like interleukin (IL)-6 and tumour necrosis factor (TNF)-α (3). Nihira et al. had performed transcriptomic and proteomic analysis on peripheral blood mononuclear cells in a Japanese cohort of DADA2 patients and reported elevated type II interferon signatures. By network analysis, the authors identified *STAT1 gene* as pivotal gene in pathogenesis of DADA2. Further, STAT1 phosphorylation in monocytes and B cells following interferon gamma stimulation was significantly higher in patients with DADA2 as compared to controls (12). Watanabe et al. performed single cell RNA sequencing in monocytes (CD14+) from DADA2 patients and healthy controls. They confirmed higher numbers of non-classical monocytes and an up regulation of M1 macrophage markers in DADA2 patients. Thus, authors suggested that high levels of IFNγ may drive the differentiation of monocytes to a M1 phenotype that leads to release of proinflammatory cytokine TNFα (13).

## 3 Spectrum of Clinical Manifestations

Since the initial description, vasculitis/vasculopathy has been the predominant phenotype seen in DADA2. However, a myriad of clinical manifestations are increasingly being reported.

### 3.1 Vasculopathy

Vasculopathy, affecting the medium and small sized arteries, is the commonest manifestation of DADA2. Clinical manifestations vary from limited cutaneous involvement to severe and fatal systemic vasculitis with multiorgan involvement. Cutaneous involvement includes livedoid rash, erythema nodosum, peripheral gangrene, ulcers and Raynaud’s phenomenon. Most common cutaneous manifestation of DADA2 is livedo racemosa. Skin biopsy often shows extensive neutrophil infiltration predominantly in interstitium, macrophage infiltration, and perivascular T lymphocytes without overt features of vasculitis.

In systemic involvement, central nervous system (CNS) is most commonly involved followed by renal and gastrointestinal (GI) systems. Hallmark of CNS vasculopathy is recurrent ischemic lacunar strokes. Other CNS manifestations include cranial nerve palsy, spastic diplegia, encephalopathy, peripheral neuropathy, sensory neural hearing loss, labyrinthitis, and cerebral atrophy. Although abdominal pain and inflammatory bowel disease are predominant GI manifestations, intestinal perforation and aneurysms in celiac and mesenteric arteries have also been reported (14). Renal involvement in patients with DADA2 is seen in form of renal artery stenosis, renal artery aneurysms, arterial hypertension, and glomerular scarring (1, 15, 16).

### 3.2 Immunodeficiency

**Figure 1:**
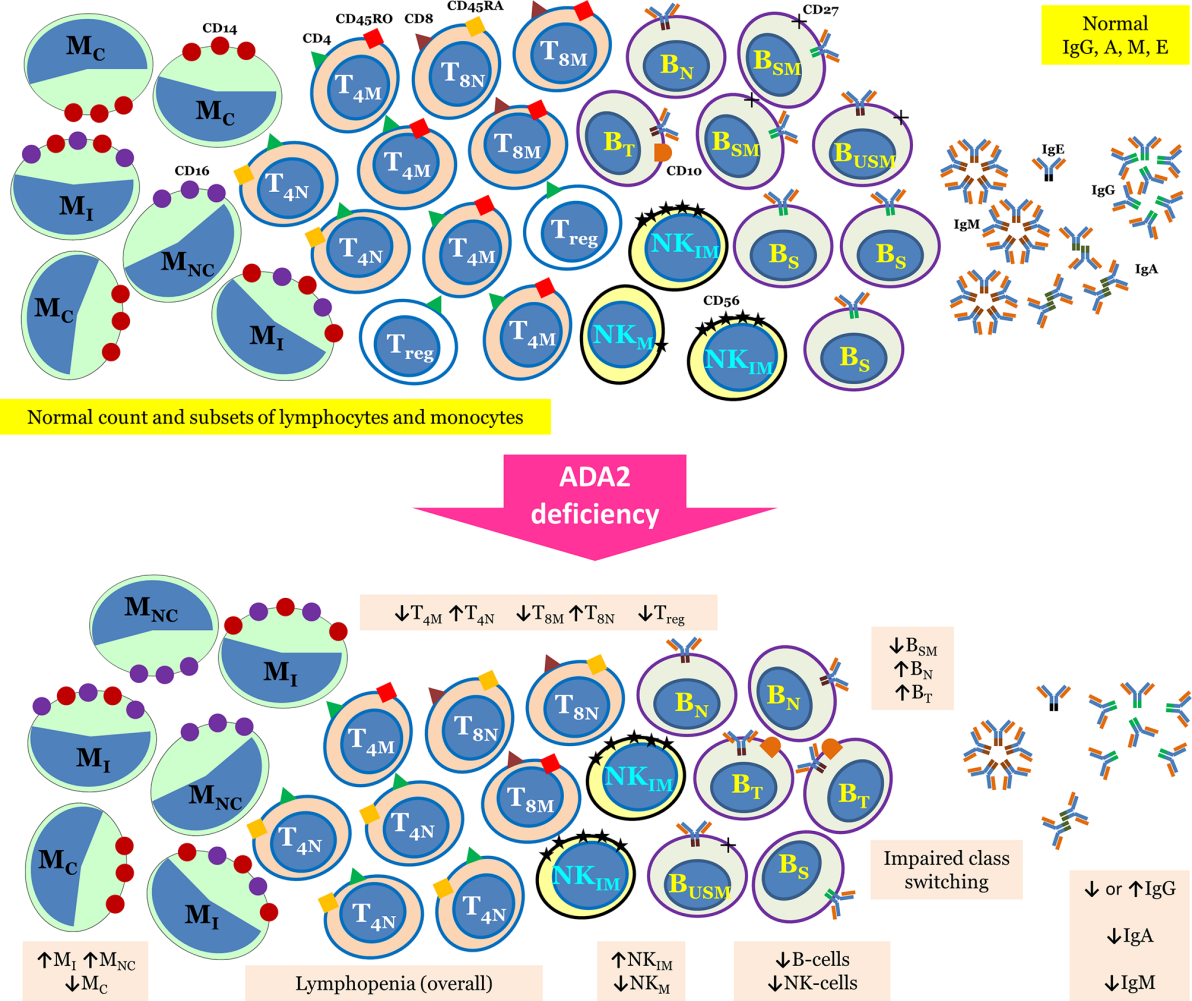
Summary of immunological abnormalities in patients with DADA2.

The initial reports of DADA2 describe this disorder as a mild immunodeficiency with reduced levels of immunoglobulin (Ig) M. Subsequently, immunological aberrations in DADA2 are being reported in a much greater detail. Up to two-thirds of patients with DADA2 may have decreased levels of either Ig isotypes, while hypogammaglobulinemia has been reported in approximately one fourth of patients (17). Impaired vaccine responses have also been reported (18). Around 10% of these patients present with B cell lymphopenia and low switched memory B cells. Overt immunodeficiency in the form of recurrent infections has been noted in 15–20% of patients (19). Clinical presentation of immunodeficiency phenotype may mimic common variable immunodeficiency (CVID). Therefore, it is prudent to consider differential diagnosis of DADA2 in patients with CVID-like immunodeficiency especially having vasculopathic manifestations (20–22). As recurrent inflammation in DADA2 may inhibit B cell differentiation and function, treatment with anti-inflammatory therapy may be beneficial in improving Ig levels (21). Tissue biopsies can also show the CVID-like phenotype with absent plasma cells (23). Besides CVID-like presentation, clinical features suggestive of a combined immunodeficiency have also been noted in DADA2. These patients have been reported with fungal infections and infections from DNA viruses including molluscum contagiosum, warts, and members of herpes virus family (24). Low numbers of natural killer (NK) cells and T cells have also been reported (18, 25). Overall, lymphopenia is reported in 15% of patients with DADA2 (17). A recent report on DADA2 patients using in-depth immunophenotyping and functional analysis of lymphocytes revealed a multitude of immunological aberrations. These include impaired class switching and differentiation of B cells, reduced memory and regulatory T cells, increased senescent T cells, diminished mucosa associated invariant T cells and invariant NKT cells, and decreased classical monocytes (26). The authors also reported arrest in B cell development in the bone marrow at the pro-B to pre-B cell stage and defect in terminal B cell differentiation. Authors have also shown that healthy heterozygous carrier state for DADA2 showed intermediate values of lymphocyte phenotypes and functions in comparison to DADA2 patients and healthy controls, further highlighting the role of heterozygous state (26). Thus, immune defect in patients with DADA2 deficiency may present with myriad immunological aberrations (26). Immunological abnormalities reported in patients with DADA2 have been summarized in **Figure 1**.

### 3.3 Hematological Manifestations of DADA2

#### 3.3.1 Bone Marrow Failure

Bone marrow hypofunction resulting in erythroblastopenia, leukopenia/neutropenia and thrombocytopenia was reported since the first descriptions of DADA2 (1). Since then, pure red cell aplasia (PRCA) or Diamond-Blackfan anemia (DBA)-like presentation is increasingly being recognized in patients with DADA2. PRCA, as the name suggests, is characterized by absence (or near absence) of red cell precursors from the bone marrow. It manifests as normocytic normochromic anemia with marked reduction in the reticulocyte response (27). DBA represents one of the congenital forms of PRCA that usually presents early in life with macrocytic (occasionally normocytic) normochromic anemia (27, 28). To date, more than 3 dozen such patients with DADA2 have been reported in the literature with DBA (29–36). As DADA2 is not commonly considered amongst the differentials of PRCA/DBA, this leads to significant diagnostic delay in some cases (36). Moreover this disorder has been described recently and possibly many cases of DBA due to DADA2 have been underreported. Majority of DADA2 patients with PRCA/DBA-like clinical features have at least one of the following clinical features, such as, benign lymphoproliferation (lymphadenopathy and hepatosplenomegaly), low IgM/IgA/IgM, or reduced B/NK/CD4-T/CD8-T cells, with or without recurrent or unusual infections. Few patients may also present with stroke and livedo reticularis which can be attributed to vasculitis or vasculopathy (29, 36–38).

Pathophysiologically, classical DBA, resulting from mutations in genes involved in ribosomal biogenesis, is associated with impaired rRNA maturation, while DBA-like illness associated with DADA2 is not (28). Although the precise mechanisms remain unknown, elevated erythroid ADA enzyme activity (especially ADA1) is seen in up to 90% patients with classical forms of DBA (28). In contrast, DADA2 is associated with normal erythrocyte ADA enzyme activity (with reduced plasma ADA2 activity) (39). In routine clinical practice, normal mean corpuscular red blood volume (MCV) in patients with DBA-like illness favours diagnosis of DADA2 over classical DBA. Besides, lack of congenital malformations, seen in ~50% of patients with classical forms of DBA, may also serve as a clue for diagnosis of DADA2 (28).

In addition to erythroid hypofunction, DADA2 may also involve other bone marrow cell lines. Of these, neutropenia is more commonly recognized (up to 10%) and has been described in numerous reports (32, 33, 40–43). Transient neutropenia has also been reported (42). Low IgM or pan-hypogammaglobulinemia, lymphopenia (40–43) has also seen in this subgroup of patients. Other than bone marrow failure syndrome manifestations recurrent fevers, oral ulcers, recurrent infections (including warts) have been reported in this subgroup of patients (32, 41–43). Besides neutropenia, a recent report from NIH on 60 patients with DADA2 describes thrombocytopenia and pancytopenia in 10% of patients (44).

ADA2 has been known to itself act as a growth factor (7, 9) with a potential to modulate secretion of other growth factors as well. Contemporary evidence also suggests both ADA1 and ADA2 enzymes to play a crucial role in development of progenitor cells in the bone marrow (45, 46). The precise contributions of growth factor or ectonucleotidase properties of ADA2 towards development of marrow cells remains to be delineated, resulting in significant gaps in understanding of pathogenesis of marrow hypofunction. A possibility of immune-related marrow hypofunction (as a result of autoimmunity) also exists (17, 46). Besides ‘central’ (i.e. marrow hypofunction) cytopenias, ‘peripheral’ cytopenias (e.g. autoimmune) may also can occur in patients with DADA2. This overlap of ‘central’ and ‘peripheral’ cytopenias may pose a significant diagnostic and therapeutic challenge for clinicians.

#### 3.3.2 Immune Cytopenias

Autoimmune cytopenia is a common presentation of DADA2. To date, more than a dozen such patients of DADA2 have been reported accompanied with predominant autoimmune cytopenia. In most of these patients the clues for etiological diagnosis have ranged from vasculopathic ulcers (30), stroke (1), low IgG/IgM or hypogammaglobulinemia (32, 44–48), recurrent infections (18, 49) including the vaccine pathogens (46). Lymphopenia, neutropenia, thrombocytopenia (Evans syndrome), lymphoproliferation with/without elevated double negative T-cells (raising a possibility of autoimmune lymphoproliferative syndrome) has also been reported in these patients (1, 18, 30, 32, 46, 49–51). In addition to expected bone marrow examination findings of erythroid hyperplasia (with reticulocytosis), features of erythroid hypoplasia or dysplasia (with reticulocytopenia) have also been described in these patients (46, 49, 50). Direct antiglobulin test was also positive in absence of overt hemolysis and PRCA in few patients (50, 52). Concomitant occurrence of AIHA and erythroblastopenia, hence, seems to be an additional haematological clinical presentation of DADA2.

#### 3.3.3 Other Haematological Manifestations

In a recent report, arthritic presentation mimicking systemic juvenile idiopathic arthritis has also been reported in DADA2 (53). Features of macrophage activation syndrome/hemophagocytic lymphohistiocytosis (MAS/HLH), haemolytic anemia (non-immune), and persistent cytopenias (with a hypercellular bone marrow) were noted in this patient (53). MAS/HLH was also been reported in the first descriptions of this disease, besides many published and unpublished observations (1, 26, 38).

### 3.4 Lymphoproliferation in DADA2

#### 3.4.1 Benign Lymphoproliferation

Benign lymphoproliferation, resulting from follicular hyperplasia and manifesting as hepatosplenomegaly and/or lymphadenopathy, is a well-recognized feature of DADA2 and is seen in about a third of all cases (3, 17). Idiopathic Castleman disease with benign lymphoproliferation as one of the cardinal clinical manifestations has also been reported in patients with DADA2 (37, 54). Besides, EBV driven non-malignant (benign) proliferation has also been reported in DADA2 (42, 52).

DADA2 has also been reported to present with autoimmune lymphoproliferative syndrome (ALPS) like phenotype (46, 51, 55) given the occurrence of both benign lymphoproliferation and autoimmune cytopenias in this disease. Differentiating DADA2 from classical ALPS may be very difficult. Patients with DADA2 are unlikely to fulfill the primary 2009 NIH ALPS criteria (i.e., patients with DADA2 would have normal apoptosis assays and lack the relevant *FAS*, *FASL*, or *CASP10* variants) (55). Besides, presence of lymphopenia and hypogammaglobulinemia favours the diagnosis of DADA2 over classical ALPS (46). Other potential subtle clues would include normal (51) (or mildly elevated) (46, 55) double-negative T-cells and normal (46) or mildly increased (55) levels of vitamin B_12_ (well below 1500 pg/mL) in patients with DADA2. Recognition of other clinical features reminiscent of DADA2 (e.g. vasculopathy/vasculitis) may also help to clinch the diagnosis.

#### 3.4.2 Malignant/Neoplasms

Malignant lymphoproliferation or neoplasms are rare in DADA2. To date, less than a dozen such patients have been reported in the literature. T-cell large granulocytic lymphocytic infiltration/leukemia (T-LGL I/L) has been reported in 2 patients (18). Both these patients also had AIHA and organomegaly concomitantly or prior to diagnosis of T-LGL I/L. Besides, decreased numbers of plasmablasts, transitional B cells, and total switched memory B cells with an increase in activated B and CD4 T-cells (HLA-DR^+^), CD8 effector memory RA cells were noted in both patients. Autoimmune neutropenia, thrombocytopenia, pan-hypogammaglobulinemia, and recurrent infections were also described during the clinical course in one of the above patients (18). Hodgkin lymphoma (HL) has been reported in 4 patients with DADA2 including a sibling pair (51, 56–58). In the siblings with HL and DADA2, lymphopenia and hepatosplenomegaly were noted before and hypogammaglobulinemia after initiation of chemotherapy for HL. Both these patients had positive anti-EBNA (Epstein-Barr virus nuclear antigen) IgG but negative anti-EBNA IgM (57). In the other 2 patients, arthritis, vasculopathy, and neutropenia were additional features of DADA2 (57, 58). Cutaneous acute myeloid leukaemia (AML) (30) and diffuse large B cell lymphoma (DLBCL) (with high EBV viral load) have also been reported in patients with DADA2 (33, 59).

## 4 Differential Diagnosis

As we have summarized, DADA2 is associated with a myriad of clinical manifestations. Hence, this disorder would be an important differential for a variety of illnesses including vasculitis/vasculopathy, inborn errors of immunity (especially humoral immunodeficiencies) including immune dysregulatory (e.g. recurrent fevers, HLH) and lymphoproliferative disorders (e.g. ALPS), PRCA/DBA, marrow dysfunction/pancytopenia, autoimmune cytopenias, and occasionally neoplasms. Although DADA2 might present with single system involvement, presence of personal or family history of relevant multisystem manifestations (e.g. lymphoproliferation and vasculitis/vasculopathy, cytopenias and hypogammaglobulinemia, and other combinations) may, by far, be the simplest clinical clue for its diagnosis.

## 5 Laboratory Diagnosis of DADA2

### 5.1 Quantification of ADA2 Enzymatic Activity

ADA2 activity can be measured by spectrophotometry or LC-MS/MS based assay in serum/plasma/tissue-culture supernatant or dried plasma spot respectively. It quantifies the adenosine-dependent generation of ammonia in the presence of erythro-9-Amino-β-hexylα-methyl-9H-purine-9-ethanol hydrochloride (EHNA), a selective inhibitor of ADA1. It is essential to perform ADA enzyme activity in addition to genetic analysis since heterozygous carriers can present with decreased enzymatic activity and clinical manifestations (38). Newer cost-effective and rapid methods for estimation of ADA2 enzyme activity may serve as a screening tool for ordering genetic testing in patients with DADA2. Utilizing such techniques, Cafaro et al. noted all patients with variant-proven DADA2 to have ADA2 enzyme activity of ≤0.06 mU/mL (60).

### 5.2 Genetics

Genetic sequencing remains the mainstay of genetic diagnosis of DADA2. Next-generation sequencing (NGS) including whole-exome sequencing is increasingly being utilized to diagnose DADA2, even when assays for ADA2 enzyme activity are not readily available (61). Given the pleiotropic manifestations of DADA2, it is important to include *ADA2* gene in various customized NGS panels used for evaluation of haematological, immunological, and rheumatological disorders. As the most common disease variants are found in exon-2 (p.G47R, p.G47A) followed by exon 3 and 4 (**Supplementary Table**), Sanger sequencing can also be employed upfront for evaluation. Few *ADA2* pathogenic variants (homozygous 800 bp duplication in exon 7), however, may not be detected by such strategies. Other techniques such as Multiplex Ligation-dependent Probe Amplification (MLPA) in combination with long-read polymerase chain reaction (PCR) sequencing need to be employed in such scenarios (55). Deep RNA sequencing can be used to evaluate the impact of novel splice variants (62).

## 6 Profile of Pathogenic Variants in *ADA2* Gene

**Figure 2:**
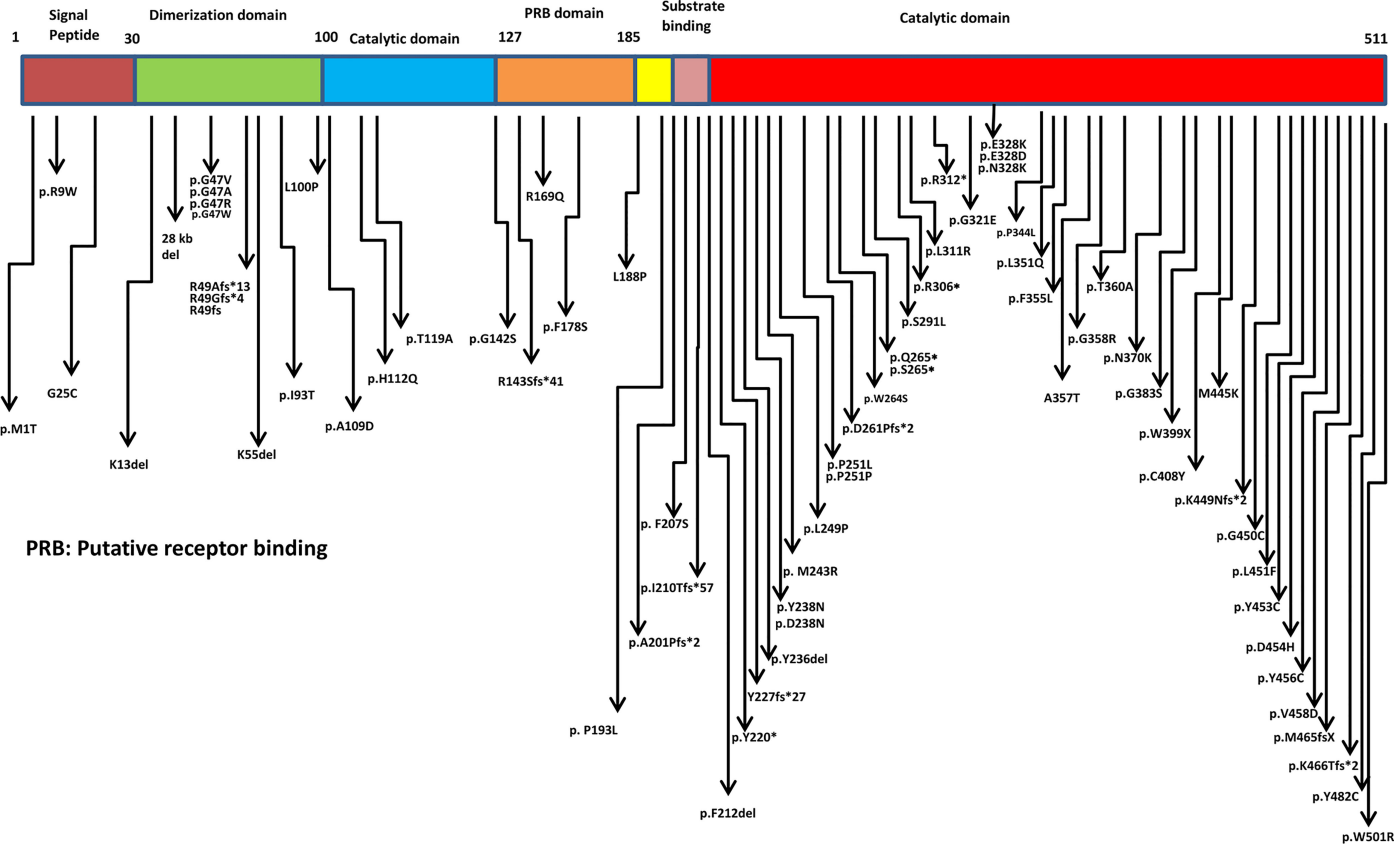
Schematic representation of mutations in ADA2 protein.

Missense, nonsense, splice site mutations, frame shift mutations, deletions, and copy number variations have been documented in the ADA2 protein with maximum clustering seen in the catalytic domain (**Figure 2** and **Supplementary Figure 1**). The most commonly reported mutations in different ancestries are p.Gly47Arg (Asian, Georgian-Jewish, Turkish), p.Gly47Ala (European Caucasian), p.Arg169Gln (European Caucasian, Dutch, Belgium, and Finnish), and p.Tyr453Cys (European Caucasian) (17). Till date, more than 100 disease causing variants has been identified in the *ADA2* genes (**Supplementary Table**). Hotspot variants for bone marrow failure, vasculitis and PRCA were R169Q, G47R and G358R respectively. The most commonly seen variation was of missense type. Other than the hotspot variant some frequently observed variations in ADA2 were G25C, G47A, R49Gfs*4, R49Afs*13, F178S, L188P, P251L, R306X, L351Q, T360A, Y453C, K466Tfs*2. Variants associated with haematological manifestations are enumerated in (**Table 1**). In general, variants leading to complete (or almost complete) loss of ADA2 enzyme activity have been associated with a predominant haematological phenotype (35).

**Table 1:** Variants associated with haematological manifestations.

S. no.	Amino acid change	cDNA position	Erythroblastopenia	Marrow dysfunction	AICs	Others
1	p.M1T	c.2T>C		+ F	+ ES	
2	p.G47R	c.139G>C				+ MAS/HLH
3	p.G47W	c.139G>T	+	+ N, F		
4	p.G47A	c.140G>C		+ P		
5	p.G47V	c.140G>T		+ N	+ HA	
6	p.R49Gfs*4	c.144delG	+			
7	p.R49Afs*13	c.143dup	+		+ HA	+ MAS/HLH
8	p.R49fs	c.144dupG	+	+ N		
9	p.I93T	c.278T>C			+ ES	
10	p.H112Q	c.336C>G	+	+ N, P		
11	p.R131Sfs*53	c.393del		+ P		
12	p.H133Lfs*44	c.396_397del			+ HA	
13	p.R169Q	c.506G>A	+	+ F, N, P	+ HA	+ MAS/HLH
14	p.F178S	c.533T>C	+	+ F		
15	p.T187P	c.559A>C			+ ES	
16	p.L188P	c.563T>C	+	+ N	+ HA, ES	
17	p.F207S	c.620T>C	+			
18	p.I210Tfs*57	c.629delT	+			
19	p.F212del	c.634_636delTTC		+ F		
20	p.Y220*	c.660C>A	+	+ P	+ HA	
21	p.Y227fs*27	c.680_681delAT	+			
22	p.E237R fs*30	c.709delC		+ N		
23	p.A247Qfs*16	c.714_738dup	+		+ HA	
24	p.V252Tfs*7	c.(753 + 168_754-229)del	+			
25	p.D261Pfs*2	c.781delinsCCATA		+ P		
26	p.S265*	c.794C>G		+ N		
27	p.R306X	c.916C>T	+			
28	p.L311R	c.932T>G	+	+ N, T		
29	p.R312*	c.934C>T		+ N		
30	p.G321E	c.962G>A	+	+ N, F		
31	p.Y353H	c.1057T>C		+ N, F		
32	p.G358R	c.1072G>A	+	+ N, F		
33	p.?	c.(1081 + 139_1082-92)del	+			
34	p.N370K	c.1110C>A	+	+ N		
35	p.W399X	c.1196G > A		+ N		
36	p.M445K	c.1334T>A	+			
37	p.K449Nfs*2	c.1346_1347insTT		+ F		
38	p.L451W	c.1352T>G	+			
39	p.L451F	c.1353G>T		+ N, F	+ HA	
40	p.Y453C	c.1358A>G		+ P, N		
41	p.D454H	c.1360G>C	+			
42	p.Y456C	c.1367A>G	+	+ N		
43	p.V458D	c.1373T>A		+ F		
44	p.M465fsX	c.1392dup	+	+ N		
45	p.K466Tfs*2	c.1397_1403delAGGCTGA	+	+ F		
46	p.Y482C	c.1445A > G	+			
47	p.(Ser483Profs*5)	c.1447_1451del		+ N	+ HA	
48	p.W501R	c.1501 T>C or T>A		+ F		
49		c.-47+2T>C	+			
50		c.1443-2T>A	+			
51		c.882-2A>G	+			
52		800-bp duplication	+			

AICs, Autoimmune cytopenias; F, Bone Marrow Failure; N, Neutropenia; P, Pancytopenia; ES, Evans Syndrome; HA, Hemolytic anaemia; HLH, Hemophagocytic lymphohistiocytosis; MAS, Macrophage Activation Syndrome; RCA, Red Cell Aplasia; DBA, Diamond-Blackfan anemia.+Presence of manifestation.

## 7 Overview of Treatment in DADA2

Treatment of patients with DADA2 primarily depends on the clinical presentation. Besides specific therapy, supportive care (e.g. wound/ulcer care, antimicrobials for infections, etc.) is also essential to ensure better outcomes.

### 7.1 Anti-Inflammatory Therapy

Corticosteroids are widely used in acute phase of the disease; however, patients often show only a modest response. Disease flares are common while tapering steroids. A steroid-refractory course has also been described (2, 30, 43). TNF blockade is the therapy of choice for vasculitic and inflammatory manifestations. Etanercept and adalimumab are the commonly employed TNF inhibitors. In resource constrained settings, thalidomide may be used as an alternative due to its anti-TNF activity. Carosi et al. have reported the effectiveness of thalidomide in controlling disease activity in 7 patients (14). IL-6 blockers (tocilizumab) are also effective in controlling inflammation; however, recurrence of stroke has been noted (12, 23, 54, 63, 64). Tocilizumab has been used successfully in the patient with Castleman disease and DADA2. IL-1 blockade has not been noted to be of significant therapeutic benefit in patients with DADA2 (65).

### 7.2 Treatment of Immuno-Hematological Manifestations

In patients with hypogammaglobulinemia, immunoglobulin replacement therapy and antibiotic prophylaxis (21, 35) are the usual treatment modalities. Hematological manifestations are usually refractory to glucocorticoids (30). Other immunosuppressive drugs (azathioprine, mycophenolate mofetil, cyclosporine and anti-thymocyte globulin) have shown variable response in conditions like PRCA and other hematological phenotypes (35, 66). Rituximab has been shown to result in a favorable response in patients with autoimmune cytopenias (32). Mild manifestations (e.g. lymphopenia) may respond to TNF-blockers (66–68); however, TNF-blockade is ineffective for treatment of severe hematological manifestations (e.g. bone marrow failure and PRCA/DBA) (33, 35).

### 7.3 Hematopoietic Stem Cell Transplantation (HSCT)

HSCT is the definitive treatment for hematological and immunological manifestations of DADA2. Hashem et al. have recently collated the multicentric experience of HSCT in 30 patients of DADA2 who underwent total 38 HSCTs. Indications for HSCT were bone marrow failure syndromes, autoimmune cytopenia, lymphoproliferation (benign or malignant) and immunodeficiency phenotypes. Overall survival after 2 years of follow-up was 97% and HSCT resolved the hematological phenotypes in all patients (33). Plasma ADA2 activity may be restored to normal as early as 2 weeks post-transplant. HSCT may also benefit vasculopathic manifestations (33).

## 8 Conclusions

DADA2 may present with diverse hematological manifestations such as DBA/PRCA, immune cytopenia, bone marrow failure syndromes, lymphoproliferation and immunodeficiency. A detailed history, comprehensive clinical examination, and basic laboratory investigations are imperative in recognizing DADA2 in such scenarios. With increasing availability and decreasing costs of NGS, genetic testing seems to be a feasible option for diagnosing DADA2 (and other inborn errors of immunity) in patients with unexplained hematological manifestations.

## Author Contributions

RP: Inception of idea, writing of initial draft of manuscript, editing and critical revision of manuscript at all stages of its production, final approval of manuscript. AB, SSh, RK, VJ, and SL: writing of initial draft of manuscript, editing and revision of manuscript at all stages of its production, review of literature. MD, AJ, PV, and DS: Contributed to editing of manuscript, review of literature. AR and SSi: Critically revision of the manuscript at all stages of its production, final approval of manuscript, and review of literature. All authors contributed to the article and approved the submitted version.

## Conflict of Interest

The authors declare that the research was conducted in the absence of any commercial or financial relationships that could be construed as a potential conflict of interest.

## Publisher’s Note

All claims expressed in this article are solely those of the authors and do not necessarily represent those of their affiliated organizations, or those of the publisher, the editors and the reviewers. Any product that may be evaluated in this article, or claim that may be made by its manufacturer, is not guaranteed or endorsed by the publisher.

## Supplementary Material

Supplementary Table 1 Variants in *DADA2* gene.

Supplementary Figure 1
*DADA2* gene sketch showing disease associated mutations in *ADA2* gene.
