# Supplementary material for: Deficiency of Human Adenosine Deaminase Type 2 – A Diagnostic Conundrum for the Hematologist
Source: Front Immunol. 2022 May 3;13:869570. doi: 10.3389/fimmu.2022.869570 (PMC9110783; doi:10.3389/fimmu.2022.869570)
Supplement: Supplementary Table 1 — Variants in DADA2 gene. [file Table_1.docx]

**Supplementary Table 1: review of published variants in *ADA2* gene associated with deficiency of human adenosine deaminase type 2**

| **S.no.** | **Reference** | **Exon** | **Amino acid change** | **cDNA position** | **Type** | **Domain** | **Ethnicity** | **Pathogenicity** | **Novelty** |
| --- | --- | --- | --- | --- | --- | --- | --- | --- | --- |
| **1** | Zhou et al (1); Nanthapisal S et al(16) | 2 | M1T | c.2T>C | Missense | Signal peptide | Caucasian | Pathogenic | No |
| 2 | Gibson,K et al(72) | 2 | p.R9W | c.25C>T | Missense | Signal Peptide | Asian | Likely Pathogenic | No |
| 3 | Gonzales  Santiago  et al(73) | 2 | K13del | c.163_165del | Nucleotides deletion | Signal peptide | Unknown | Likely Pathogenic | No |
| 4 | Meyts I et al(17) | Inclu 5’UTR and E-1 | 28 kb deletion | Deletion | 28 kb deletion | 5‘UTR | European Caucasian | Pathogenic | No |
| 5 | Meyts I et al (15) | 2 | G25C | c.73G>T | Missense | Signal Peptide | Tunisia / Maghrebian | Likely pathogenic | No |
| 6 | Nanthpisal S et al (16);  Meyts I et al(17) | 5 Flanking |  | c.-114delC | Frameshift | Dimerization | Caucasian |  | Yes |
| 7 | Navon et a (2).;  Zhou et al (1);  Van Eyck  et al (23);  Garg et al (67);  Belot et al (74);  Batu et al (84). | 2 | G47R | c.139G>A | Missense | Dimerization | Georgian;  Turkish; NA;  Turkish;  Asian; Turkish | Pathogenic | No |
| 8 | Nanthpisal S et al (16) | 2 | G47R | c. 139G>C | Missense | Dimerization | Indian/Asian | Not classified | No |
| 9 | Zhou et al (1). | 2 | G47A | c.140G>C | Missense | Dimerization | European Caucasian | Pathogenic | No |
| 10 | Navon et al. (2) | 2 | G47V | c.140G>T | Missense | Dimerization | Turkish | Likely pathogenic | No |
| 11 | Göschl L & al. J Clin Immunol, 2020 Jan(75) | 2 | G47W | c.139G>T | Missense | Dimerization | German+Italian) Mixed | Likely Pathogenic | No |
| 12 | Nanthpisal S et al(16) | 2 | p.R49Gfs*4 | c.144delG | Frameshift | Dimerization | Italy | Pathogenic | No |
| 13 | Hashem H (36) | 2 | p.R49fs | c.144dupG | Frameshift | Dimerization |  | Pathogenic | No |
| 14 | Meyts I et a (15)l; Ben-Ami T et al (42) | 2 | R49Afs*13 | c.143 dup | Insertion, Frameshift nonsense | Dimerization | Caucasian, Israel | Likely pathogenic | No |
| 15 | Meyts I et al(15) | 2 | K55del | c.163_165del | Nucleotides deletion | Dimerization | Unknown | Likely pathogenic | No |
| 16 | Zhou et al.(1) | 2 | I93T | c.278T>C | Missense | Dimerization | Caucasian,United Kingdom | Likely pathogenic | No |
| 17 | Zhou et al (1) | 3 | A109D | c.326C>A | Missense | Catalytic domain | Caucasian/United States | Pathogenic | No |
| 18 | Zhou et al (1) | 3 | H112Q | c.336C>G | Missense | Catalytic domain | United States Caucasian | Pathogenic | No |
| 19 | Bras et al (76) (ENST00000262607.3) | 3 | T120A | c.358A>G | Missense | Catalytic domain | European Caucasian |  | No |
| 20 | Hashem,H and et al(36) | 3 | **p.R131Sfs** | **c.3936delG** | Deletion | PRB | Unknown | Likely Pathogenic | No |
| 21 | [Ferriani](https://pubmed.ncbi.nlm.nih.gov/?term=Ferriani+MPL&cauthor_id=33493352) MPL et al (48) | 3 | p.H133Lfs*44 | c.396_397del | Deletion | PRB | Brazil/Unknown | Likely Pathogenic | No |
| 22 | Meyts I et al(15) | 3 | R143Sfs*41 |  | Frameshift | PRB | Caucasian | Pathogenic | No |
| 23 | Bras et al.(76) | 3 | G142S | c.424G>A | Missense | Catalytic domain | Portugal | Likely Pathogenic | No |
| 24 | Navon et al (2);  Van Montfrans  et al (30);  Van Eyck et al (23);  Belot et al(74);  Westendorp et al(77);  Van Schepp  et al(20);  Nanthpisal S et al(16) | 3 | R169Q | c.506G>A | Missense | PRB | European Caucasian; NA; African; European Caucasian; European Caucasian | Pathogenic | No |
| 25 | Meyts I et al(15) | 3 | F178S | c.533T>C | Missense | PRB | Israel/Caucasian | Likely Pathogenic | No |
| 26 | [Gianluca Dell'Orso](https://pubmed.ncbi.nlm.nih.gov/?term=Dell%27Orso+G&cauthor_id=34721429)  et al (47) | 4 | T187P | c.559A>C |  | PRB | Italy | Likely Pathogenic | No |
| 27 | Meyts I et al(15) | 4 | L188P | c.563T>C | Missense | PRB | Germany | Likely Pathogenic | No |
| 28 | Belot et al.(74) | 4 | P193L | c.578C>T | Missense | Catalytic domain | France/Caucasian | Likely Pathogenic | No |
| 29 | Ozen, S et al(38) | 4 | p.F207S | c.620T>C | Missense | Catalytic domain | Italy/Caucasian | Not classified | No |
| 30 | Ozen, S et al (38) | 4 | p.I210Tfs*57 | c.629delT | Deletion Frameshift nonsense | Catalytic domain | Unknown | Likely Pathogenic | No |
| 31 | Tull,T et al (78) | 4 | p.F212del | c.634_636delTTC | Deletion | Catalytic domain | Unknown | Likely Pathogenic | No |
| 32 | Meyts I et al(15) | 4 | Y220* | c.660C>A | Nonnsense | Catalytic domain | Caucasian | Likely pathogenic | No |
| 33 | Ozen, S et al(38) | 4 | Y227fs*27 | c.680_681delAT | Deletion | Catalytic domain | Unknown | Likely Pathogenic | No |
| 34 | Meyts I et al(15) | 4 | Y236del | c.706_708 del | Nucleotide deletion | Catalytic domain | Japan/Asian | Likely Pathogenic | No |
| 35 | **Hashem H and et al(36)** | 4 | p.E237R fs*30 | c.709delC | Deletion | Catalytic domain | Unknown | Likely Pathogenic | No |
| 36 | Rama M et al(79) | 4 | p.D238N | c.712G>A | Missense | Catalytic domain | France / *Maghrebian* | Likely Pathogenic | No |
| 37 | Schepp  et al.(20) | 4 | M243R | c.728T>G | Missense | Catalytic domain | Unknown | Likely Pathogenic | No |
| 38 | [Albalawi](https://www.ncbi.nlm.nih.gov/pubmed/?term=Albalawi%20R%5BAuthor%5D&cauthor=true&cauthor_uid=34845942) R et al (51) | 4 | A247Qfs*16 | c.714_738 dup | Frameshift | Catalytic domain | Saudi | Likely Pathogenic |  |
| 39 | Meyts I et al (15) | 4 | L249P | c.746T>C | Missense | Catalytic domain | Italy/Unknown | Not classified |  |
| 40 | Navon et al (2);  Nanthapisal S et al(16) | 4 | P251L | c.752C>T | Missense | Catalytic domain | Germany/Caucasian; Iraqi | Likely Pathogenic | No |
| 41 | Meyts I et al(15) | 4 | P251P | c.753G>A | Splicing mutation | Catalytic domain | France/Caucasian | Likely Pathogenic | No |
| 42 | Hashem H et al (36) | Intron 4 | p.V252Tfs*7 | c.(753 + 168_754-229)del | Deletion(Del E5-6-7) | Catalytic domain | Unknown/France | Pathogenic | No |
| 43 | Meyts I et al(15); Ben-Ami T et al(42) | 5 | D261Pfs*2 | c.781delinsCCATA | Frameshift | Catalytic domain | Israel/Caucasian | Likely Pathogenic | No |
| 44 | Navon et al (2) | 5 | W264S | c.791G>C | Missense | Catalytic domain | Turkish | Likely Pathogenic | No |
| 45 | Hashem H et al (36) | 5 | p.S265* | c.794C>G | Nonsense | Catalytic domain | Caucasian | Likely Pathogenic | No |
| 46 | Meyts I et al (15) | 5 | S291L | c.872C>T | Missense | Catalytic domain | France / *Maghrebian* | Likely Pathogenic | No |
| 47 | Garg et al (67) | 6 | R306X | c.916C>T | Nonsense | Catalytic domain | Turkish | Pathogenic | No |
| 48 | Meyts I et al (15) | 6 | L311R | c.932T>G | Missense | Catalytic domain | Finland/Caucasian | Likely Pathogenic | No |
| 49 | Meyts I et al (15); Caorsi et al (14) | 6 | R312* | c.934C>T | Nonsense | Catalytic domain | Italy | Likely Pathogenic | No |
| 50 | Meyts I et al (15) | 6 | G321E | c.962G>A | Missense | Catalytic domain | Unknown | Likely Pathogenic | No |
| 51 | Meyts I et al (15);  Keer N et al (80). | 7 | E328K | c.982G>A | Missense | Catalytic domain | Unknown | Likely Pathogenic | No |
| 52 | Meyts I et al (15); Caorsi et al (14) | 7 | E328D | c.984G>C | Missense | CatalytiC  domain | Italy | Likely Pathogenic | No |
| 53 | Gonzales  Santiago  et al (73) ((NM_00128227.1)) | 7 | N328K | c.984C>A | Missense | Catalytic domain | European Caucasian |  | No |
| 54 | Meyts I et al (15); Caorsi et al (14) | 7 | P344L | c.1031C>T | Missense | Catalytic domain | Italy | Likely Pathogenic | No |
| 55 | Gibson K et al (72) | 7 | p.L351Q | c.1052T>A | Missense | Catalytic domain | Caucasian | Likely Pathogenic | No |
| 56 | Ghurye,R et al (40) | 7 | Y353H | c.1057T>C | Missense | Catalytic domain | Unknown | Likely Pathogenic | No |
| 57 | Meyts I et al (15), Keer N et al (80) | 7 | F355L | c.1065C>A | Missense | Catalytic domain | Unknown | Likely Pathogenic | No |
| 58 | Gibson K et al (72) | 7 | A357T | c.1069G>A | Missense | Catalytic domain | Asian | Likely Pathogenic | No |
| 59 | Meyts I et al (15) | 7 | G358R | c.1072G>A | Missense | Catalytic domain | Caucasian | Likely Pathogenic | No |
| 60 | Meyts I et al (15); Bras,J et al(76) | 7 | T360A | c.1078A>G | Missense | Catalytic domain | Portugal | Likely Pathogenic | No |
| 61 | Meyts I et al (15); Gonzalez Santiago et al (73). | 8 | N370K | c.1110C>A | Missense | Catalytic domain | Unknown | Likely Pathogenic | No |
| 62 | Meyts I et al (15); Bras,J et al(76) | 8 | G383S | c.1147G>A | Missense | Catalytic domain | Portugal | Likely Pathogenic | No |
| 63 | Barzaghi,F et al (57) | 8 | p.W399X | c.1196G>A | Nonsense | Catalytic domain | Caucasian | Likely Pathogenic | No |
| 64 | Meyts I et al (5); Skrabl-Baumgartner,A et al (82) | 8 | C408Y | c.1223G>A | Missense | Catalytic domain | Caucasian | Pathogenic | No |
| 65 | [Ulirsch](https://www.ncbi.nlm.nih.gov/pubmed/?term=Ulirsch%20JC%5BAuthor%5D&cauthor=true&cauthor_uid=30503522) JC et al (34) | 8 | M445K | c.1334T>A | Missense | Catalytic domain |  | Pathogenic | No |
| 66 | Lee PY et al (35) | 9 | p.K449Nfs* | c.1346_1347insTT | Insertion TT | Catalytic domain | Unknown | Not classified | No |
| 67 | Meyts I et al (15) | 9 | G450C | c.1348G>T | Missense | Catalytic domain | France/Caucasian | Likely Pathogenic | No |
| 68 | Meyts I et al(15) | 9 | L451F | c.1353G>T | Missense | Catalytic domain | Israel/Caucasian | Likely Pathogenic | No |
| 69 | Ekinci, RMK, et al (50) | 9 | L451W | c.1352T>G | Missense | Catalytic domain | Syrian Arab Republic | Likely Pathogenic | No |
| 70 | Zhou et al(1); Nanthpisal S et al(16) | 9 | Y453C | c.1358A>G | Missense | Catalytic domain | European Caucasian | Pathogenic | No |
| 71 | Ozen S et al (38) | 9 | p.D454H | c.1360G>C | Missense | Catalytic domain | Unknown | Likely Pathogenic | No |
| 72 | Lamprecht,P et al (83) | 9 | p.Y456C | c.1367A>G | Missense | Catalytic domain | Unknown | Likely Pathogenic | No |
| 73 | Meyts I et al (15) | 9 | V458D | c.1373T>A | Missense | Catalytic domain | Caucasian | Likely Pathogenic | No |
| 74 | Ozen S et al (38) | 9 | p.M465fsX | c.1392dup | Duplication | Catalytic domain | Unknown | Likely Pathogenic | No |
| 75 | Lee PY et al (35) | 9 | p.K466Tfs*2 | c.1397_1403delAGGCTGA | Deletion | Catalytic domain | Unknown | Not classified | No |
| 76 | Meyts I et al(15); Ozen S et al (38) | 10 | p.Y482C | c.144  5A>G | Missense | Catalytic domain | Unknown | Likely Pathogenic | No |
| 77 | [Albalawi](https://www.ncbi.nlm.nih.gov/pubmed/?term=Albalawi%20R%5BAuthor%5D&cauthor=true&cauthor_uid=34845942) R et al (51) | 10 | S483Pfs*5 | c.1447_1451del | Deletion | Catalytic domain | Unknown | Likely Pathogenic | No |
| 78 | Meyts I et al(15) | 10 | W501R | c.1501 T>C or T>A | Missense | Catalytic domain |  |  | No |
| 79 | Alsultan A et al (52); Meyts I et al(15) | Intron 5 |  | c.882-2A>G | Splicing mutation | Catalytic | Asian | Likely Pathogenic | No |
| 80 | Meyts I et al (15) | Intron 1 |  | c.-47+2T>C | Nucleotide deletion | Signal peptide | Unknown | Likely Pathogenic | No |
| 81 | Meyts I et al (15) | 2 |  | c.138-144delG | Frameshift | Dimerization |  |  |  |
| 82 | Meyts I et a(15)l; Schepp,J et al (20) | Intron 3 |  | c.542+1G>A | Splicing mutation |  | Unknown | Likely Pathogenic | No |
| 83 | Meyts I et al (15), Schepp J et al (20) | Intron 6 |  | c.972+3A>G | Splicing mutation | Catalytic domain | Unknown | Likely Pathogenic | No |
| 84 | Meyts I et al(15); Schepp,J et al (20) | Intron 6 |  | c.973-1G>A | mRNA Splicing mutation | Catalytic domain | Unknown | Likely Pathogenic | No |
| 85 | Meyts I et al (15) | Intron 6 |  | c.973-2A>G | Splicing mutation | Catalytic domain | Netherland/Caucasian | Likely Pathogenic | No |
| 86 | Hashem, H et al (36) | Intron7 |  | c.(1081 + 139_1082-92)del | Deletion | Catalytic domain | Unknown | Likely Pathogenic | No |

**Additional references for Supplementary table 1:**

1. Miano M, Guardo D, Grossi A, Palmisani E, Fioredda F, Terranova P, Lupia M, Cappelli E, Dell'Orso G, Lanciotti M, Ceccherini I. Genetic Screening of Patients with Evans Syndrome: A Single Centre Analysis. Blood. 2021;138(Supplement 1):4198. doi: <https://doi.org/10.1182/blood-2021-151450>.
2. [Gibson](https://pubmed.ncbi.nlm.nih.gov/?term=Gibson+KM&cauthor_id=31008556) KM , [Morishita](https://pubmed.ncbi.nlm.nih.gov/?term=Morishita+KA&cauthor_id=31008556) KA , [Dancey](https://pubmed.ncbi.nlm.nih.gov/?term=Dancey+P&cauthor_id=31008556) P ,et al. Identification of Novel Adenosine Deaminase 2 Gene Variants and Varied Clinical Phenotype in Pediatric Vasculitis. Arthritis Rheumatol 2019;71(10):1747-1755.
3. Gonzalez Santiago TM, Zavialov A, Saarela J, et al. Dermatologic features of ADA2 deficiency in cutaneous polyarteritis nodosa. JAMA Dermatol. 2015;1.
4. Belot A, Wassmer E, Twilt M, et al. Mutations in CECR1 associated with a neutrophil signature in peripheral blood. Pediatr Rheumatol Online J. 2014;12:44. 9.
5. [Goschl](https://pubmed.ncbi.nlm.nih.gov/?term=G%C3%B6schl+L&cauthor_id=31686313) L , [Winkler](https://pubmed.ncbi.nlm.nih.gov/?term=Winkler+S&cauthor_id=31686313) S , Dmytrus J et al. [Unreported Missense Mutation in the Dimerization Domain of ADA2 Leads to ADA2 Deficiency Associated with Severe Oral Ulcers and Neutropenia in a Female Somalian Patient-Addendum to the Genotype-Phenotype Puzzle.](https://www.ncbi.nlm.nih.gov/pubmed/31686313) J Clin Immunol 2020;40(1):223-226.
6. Bras J, Guerreiro R, Santo GC. Mutant ADA2 in vasculopathies. N Engl J Med. 2014;371(5):478–80.
7. Westendorp WF, Nederkoorn PJ, Aksentijevich I et al. Unexplained earlyonset lacunar stroke and inflammatory skin lesions: consider ADA2 deficiency. Neurology. 2015;84(20):2092–3.
8. Tull TJ, Martin B, Spencer J et al. Sneddon syndrome associated with two novel ADA2 gene mutations. Rheumatology. 2019;59:1448–50.
9. [Rama](https://www.ncbi.nlm.nih.gov/pubmed/?term=Rama%20M%5BAuthor%5D&cauthor=true&cauthor_uid=29681619) M,  [Duflos](https://www.ncbi.nlm.nih.gov/pubmed/?term=Duflos%20C%5BAuthor%5D&cauthor=true&cauthor_uid=29681619) C,  [Melki](https://www.ncbi.nlm.nih.gov/pubmed/?term=Melki%20I%5BAuthor%5D&cauthor=true&cauthor_uid=29681619) I et al. A decision tree for the genetic diagnosis of deficiency of adenosine deaminase 2 (DADA2): a French reference centres experience. Eur J Hum Genet 2018.
10. Keer N, Hershfield M, Caskey T et al. Novel compound heterozygous variants in CECR1 gene associated with childhood onset polyarteritis nodosa and deficiency of ADA2. Rheumatology (Oxford). 2016;55(6):1145–7.
11. [Barzaghi](https://pubmed.ncbi.nlm.nih.gov/?term=Barzaghi+F&cauthor_id=30692987) F ,  [Minniti](https://pubmed.ncbi.nlm.nih.gov/?term=Minniti+F&cauthor_id=30692987) F ,  [Mauro](https://pubmed.ncbi.nlm.nih.gov/?term=Mauro+M&cauthor_id=30692987) M et al. ALPS-Like Phenotype Caused by ADA2 Deficiency Rescued by Allogeneic Hematopoietic Stem Cell Transplantation. Front Immunol 2019;9:2767.
12. [Baumgartner](https://pubmed.ncbi.nlm.nih.gov/?term=Skrabl-Baumgartner+A&cauthor_id=28830446) AS ,  [Plecko](https://pubmed.ncbi.nlm.nih.gov/?term=Plecko+B&cauthor_id=28830446) B, [Schmidt](https://pubmed.ncbi.nlm.nih.gov/?term=Schmidt+WM&cauthor_id=28830446) WM^s^et al. Autoimmune phenotype with type I interferon signature in two brothers with ADA2 deficiency carrying a novel CECR1 mutation. Pediatr Rheumatol Online J. 2017;15(1):67
13. Lamprecht P, Humrich JY, Diebold I et al. Diagnosis of deficiency of adenosine deaminase 2 with early onset polyarteritis nodosa in an adult patient with a novel compound heterozygous CECR1 mutation. Clin Exp Rheumatol 2018;36:S111(2):177.
14. Batu ED, Karadag O, Taskiran EZ, et al. A case series of adenosine deaminase 2-deficient patients emphasizing treatment and genotype-phenotype correlations. J Rheumatol. 2015;42(8):1532–4.
