# Supplementary figures and images for: Deficiency of Human Adenosine Deaminase Type 2 – A Diagnostic Conundrum for the Hematologist
Source: Front Immunol. 2022 May 3;13:869570. doi: 10.3389/fimmu.2022.869570 (PMC9110783; doi:10.3389/fimmu.2022.869570)

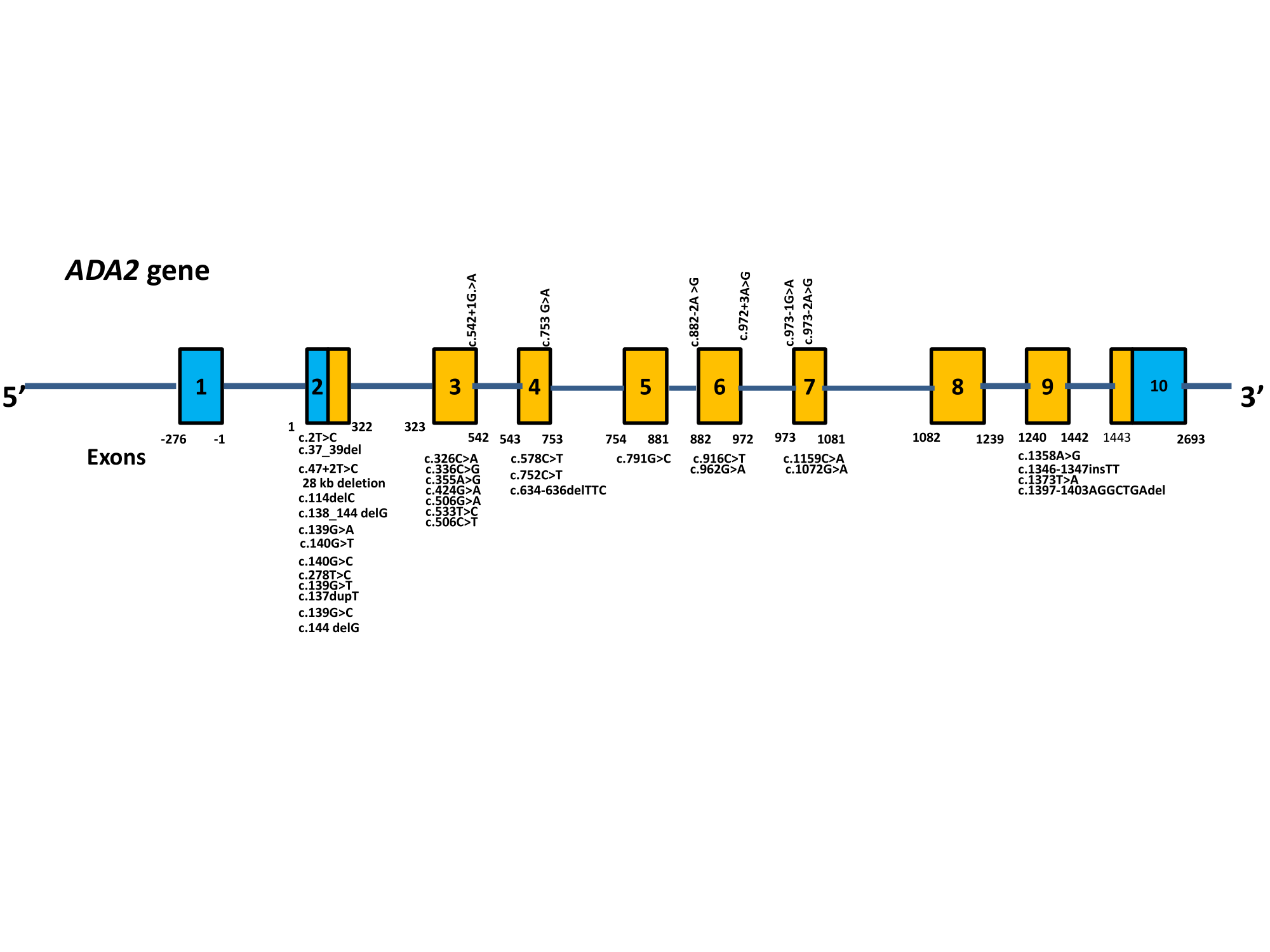

Supplement: Supplementary Figure 1 — DADA2 gene sketch showing disease associated mutations in ADA2 gene. [file Image_1.tiff]
